# Phenotypic Physiological and Metabolomic Analyses Reveal Crucial Metabolic Pathways in Quinoa (*Chenopodium quinoa* Willd.) in Response to PEG-6000 Induced Drought Stress

**DOI:** 10.3390/ijms26062599

**Published:** 2025-03-13

**Authors:** Qinghan Bao, Yang Wu, Huishi Du, Yang Wang, Yongping Zhang

**Affiliations:** 1 College of Life Sciences, Jilin Normal University, Siping 136000, China; baoqinghan@jlnu.edu.cn; 2 College of Agriculture, Inner Mongolia Agricultural University, Hohhot 010018, China; wuyang@imau.edu.cn; 3 College of Geographic Sciences and Tourism, Jilin Normal University, Siping 136000, China; duhs@jlnu.edu.cn

**Keywords:** quinoa, drought stress, phenotypic, physiological, metabolomics

## Abstract

Drought stress seriously threatens human food security, and enhancing crops’ drought tolerance is an urgent problem to be solved in breeding. Quinoa is known for its high nutritional value and strong drought tolerance, but its molecular mechanism in response to drought stress is still unclear. In this study, we used drought-tolerant (D2) and drought-sensitive (ZK1) quinoa varieties, and PEG-6000 was used to simulate drought stress in quinoa seedlings. Phenotypic and physiological biochemical indicators were measured during the seedling stage, and LC-MS was used for a metabolite analysis of drought stress to explore the drought tolerance mechanism of quinoa under drought stress. With the intensification of drought stress, chlorophyll content gradually increased, and D2 reached its maximum at W4, an increase of 49.85% compared with W1. The total chlorophyll content, photosynthesis rate, and stomatal conductance of ZK1 were significantly lower than D2 under moderate and severe drought stress. Metabolomic results showed that a total of 1295 positive ion mode (pos) metabolites and 914 negative ion mode (neg) metabolites were identified. Of these, 12(R)-HETE, phosphatidylcholine, monogalactose diester (MGDG), and stachyose up-regulated expression under drought stress. Kyoto Encyclopedia of Genes and Genomes (KEGG) analysis showed that unsaturated fatty acid biosynthesis and glycerophospholipid metabolism pathways were significantly enriched. In summary, our results elucidate that quinoa responds to drought stress by accumulating chlorophyll and sugars, activating unsaturated fatty acid metabolism, and protecting the photosynthetic system. These findings provide new insights for the breeding of drought-tolerant quinoa varieties and the study of drought tolerance mechanisms.

## 1. Introduction

Drought stress is a serious threat to human food security and a major constraint on the development of global agricultural production [1,2,3,4]. With population growth, urban expansion, desertification, and soil salinization [5,6], land resources are becoming increasingly scarce. Therefore, improving the drought tolerance of crops is one of the most pressing problems to be addressed in current breeding efforts [7].

Under drought stress, the development of roots and leaves is slowed down, resulting in a reduction in biomass [8]. To adapt to the water deficit environment, plants adjust cellular osmotic pressure by changing the characteristics of the root system, with strategies such as longer and thinner primary roots, more fibrous roots, and increased total root area [9]; increasing stomatal density and altering stomatal density and stomatal aperture; accumulating soluble sugars, proline, and ions to regulate cellular osmotic pressure [10]; and accumulating chlorophyll to enhance photosynthesis [11]. When plants are subjected to abiotic stress, they respond by signaling and expressing related genes such as bZIP, AP2/ERE, MYB/MYC [12], WRKY [11,13], NAC [14], and other families of transcription factors with important regulatory roles.

Metabolomics involves the study of the composition of and dynamic changes in all small-molecule metabolites in plants [15]. It is a comprehensive approach that closely correlates with phenotypic traits, reveals gene functions, and effectively elucidates the growth and adaptation mechanisms of plants in adverse environments [16]. It has been extensively used to study plant responses to abiotic stresses. Metabolomics is being used to characterise the stress responses of drought-susceptible and drought-tolerant varieties and to identify potential stress-related biomarkers. Drought stress often coexists with osmotic stress, where amino acids act as osmotic defences. The importance of other metabolites, including organic acids, sugars, and phenolics, in abiotic stress has also been established [17]. Under drought stress in barley, the levels of L-proline, mannitol, and other osmotic defenses increase. In DS and DT wild genotypes, α-ketoglutarate levels decreased [18]. Quinoa responds to drought stress by accumulating polypeptides, organic acids, and amino acids, thereby increasing its osmoregulatory capacity [19]. Arabidopsis leaves produce abscisic acid (ABA) under drought stress, which accumulates amino acids, polyamines, and raffinose under the action of ABA. Drought results in the accumulation of more cyclic amino acids such as proline, tryptophan, phenylalanine, and histidine in maize leaves, while pyruvate and quinic acid decrease [20,21].

Quinoa (*Chenopodium quinoa* Willd.) has a very high nutritional value [22,23,24]. In addition, the high quality of the protein and the well-balanced amino acid profile are the most outstanding nutritional characteristics of quinoa [21]. In addition, quinoa is well adapted to different agro-ecological environments, it can grow in marginal lands where nutrients are scarce, it can grow normally in saline alkali soil and infertile soil, and it has a certain tolerance to stress conditions such as frost, drought, and strong solar radiation [25,26,27]. Quinoa has high drought tolerance. Quinoa yields remain stable in the semi-arid desert regions of Chile, Peru, and Bolivia, where annual rainfall is less than 300 mm [28,29,30]. Drought stress promotes root growth in drought-tolerant quinoa, with higher root length, root diameter, and root number than drought-sensitive varieties. Under extreme drought conditions, quinoa achieves regulation of water absorption by controlling stomatal switching to influence the transpiration rate [31,32]. ABA plays a crucial role in the response of quinoa to drought stress. Under drought stress, quinoa stomata close while xylem ABA levels increase, maintaining high water use efficiency. ABA is thought to regulate the expansion pressure of stomatal defence cells to maintain cell water content, which is one of the response mechanisms of quinoa to drought stress [33,34,35,36]. At present, there is limited research on the metabolic regulation mechanism of drought tolerance in quinoa. In our study, we used different drought tolerant quinoa varieties, measured their phenotypes and physiological and biochemical indicators, and identified the changes in metabolites under drought stress using metabolomics (LC-MS) to explore the response mechanism of quinoa under drought stress. The results of this study will help us to better understand the metabolic changes in quinoa under drought stress conditions and provide a theoretical basis for the discovery of drought-tolerant breeds of quinoa.

## 2. Results

### 2.1. Effect of Drought Stress on Growth Characteristics

**Figure 1:**
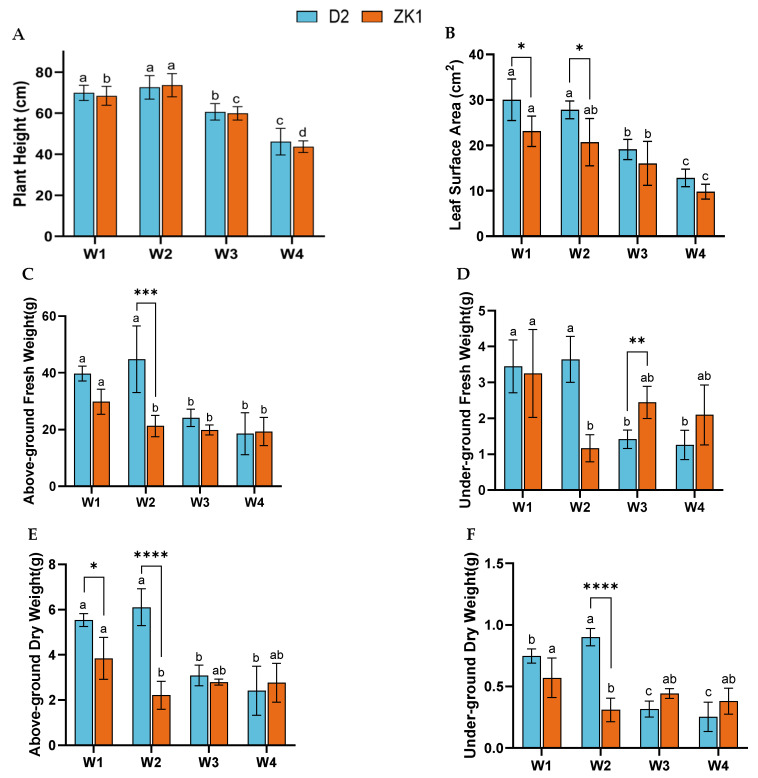
Effects of drought stress on the seedling height and biomass of quinoa varieties with different drought tolerance. The growth of quinoa seedlings under drought stress: (**A**) plant height, (**B**) leaf surface area, (**C**) aboveground fresh weight, (**D**) underground fresh weight, (**E**) aboveground dry weight, and (**F**) underground dry weight. Significant differences (*p* < 0.05) are represented by different lowercase letters. Data are expressed as means ± SD of three independent experiments. Different letters indicate statistically significant differences (*p* < 0.05) among treatments. ****, ***, **, and * indicate significant differences: (*p* < 0.0001), (*p* < 0.001), (*p* < 0.01), and (*p* < 0.05).

The plant height of D2 and ZK1 increased and then decreased with increasing drought stress. Compared with the control (W1), D2 and ZK1 plant height increased by 3.82% and 6.90% under mild stress (W2), respectively. Under moderate stress (W3) and severe stress (W4), plant height significantly decreased (*p* < 0.05), and D2 and ZK1 decreased by 31.75% and 37.00% under W4 stress (Figure 1A), respectively.

Drought stress affects leaf growth, and the leaf area under W3 and W4 stress was significantly lower than under W1 (*p* < 0.05) (Figure 1B). The leaf area of D2 and ZK1 decreased by 41.83% and 58.53%, respectively, under W3, and by 39.05% and 62.74% respectively under W4. The biomass (fresh weight, dry weight) of D2 increased under W2 stress, while W3 and W4 decreased it. The aboveground fresh weight and underground fresh weight under W2 increased by 12.78% and 5.51%, respectively. The aboveground dry weight and underground dry weight under W2 increased by 10.29% and 20.00%, respectively (Figure 1E,F). ZK1 gradually decreased with increasing stress level, and the aboveground dry weight of D2 and ZK1 decreased by 53.27% and 35.31%, respectively, while the underground dry weight decreased by 63.48% and 35.38%, respectively. In summary, drought stress has a greater impact on ZK1 in terms of the growth indicators.

### 2.2. Photosynthetic Indexes Under Drought Stress

**Figure 2:**
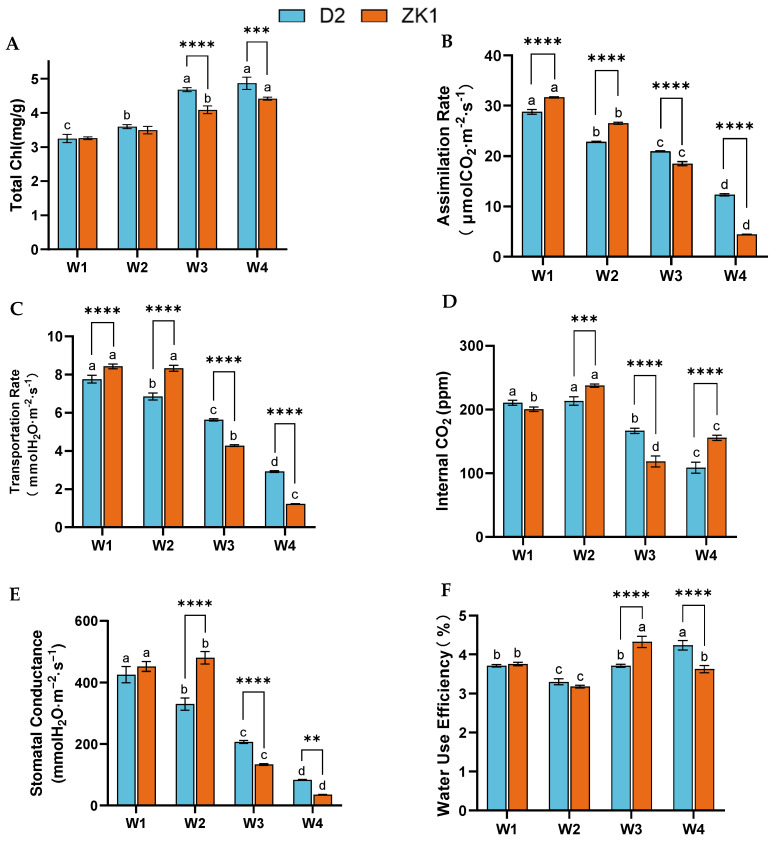
Effects on the photosynthetic parameters of quinoa with different drought tolerance. (**A**) Total Chl, (**B**) assimilation rate, (**C**) transpiration rate, (**D**) intercellular CO_2_ concentration, (**E**) stomatal conductance, and (**F**) water use efficiency. Significant differences (*p* < 0.05) are represented by different lowercase letters. Data are expressed as the means ± SD of three independent experiments. Different letters indicate statistically significant differences (*p* < 0.05) among treatments. ****, ***, and ** indicate significant differences: (*p* < 0.0001), (*p* < 0.001), and (*p* < 0.01).

The chlorophyll content of D2 and ZK1 gradually increased with increasing drought stress (Figure 2A). D2 reached the maximum chlorophyll under W4 stress, which increased by 49.85% compared with the control under W1, and the chlorophyll content of D2 was significantly higher than that of ZK1 under W3 and W4. The comparison between groups revealed that the chlorophyll content of ZK1 was significantly lower than that of D2 under W3 and W4.

Drought stress has a significant impact on the photosynthetic parameters of quinoa (Figure 2). The changes in the net photosynthetic rate (Pn) (Figure 2B) and transpiration rate (Tr) (Figure 2C) of D2 and ZK1 show the same trend, gradually decreasing with increasing stress. Under the stress of W2, W3, and W4, the Pn of D2 significantly decreased by 20.51%, 27.35%, and 57.14%, while the Pn of ZK1 significantly decreased by 16.22%, 41.51%, and 85.99%, respectively. Under W3 and W4 conditions, the decrease in Tr of ZK1 (49.23%) was greater than that of D2 (27.45%). Comparing the groups showed that both Pn and Tr were significantly higher in D2 compared with ZK1 under drought stress. This indicates that drought stress has a greater impact on ZK1 than D2.

Under W2 stress, the intercellular CO_2_ concentration (Ci) of D2 was not significantly different from that of W1 (Figure 2D), and it was significantly decreased (*p* < 0.05) under W3 and W4 conditions, with a reduction of 20.89% and 48.12%, respectively. ZK1 was the lowest under W3, with a reduction of 40.83%.

Under W2 (mild drought), the stomatal conductance (Gs) of ZK1 slightly increased compared with the control W1 (Figure 2E). W3 (moderate) and W4 (severe stress) had a greater effect on the Gs of all quinoa varieties, and the Gs of D2 and ZK1 decreased to the lowest under the W4 (severe stress) condition, which decreased by 80.47 and 92.18%, respectively, compared with the control W1. The water use efficiency (WUE) (Figure 2F) of D2 and ZK1 decreased and then increased with increasing drought stress in the test quinoa, and in ZK1, WUE decreased to the minimium under the W4 stress condition.

### 2.3. Antioxidant Enzyme Activity Under Drought Stress

**Figure 3:**
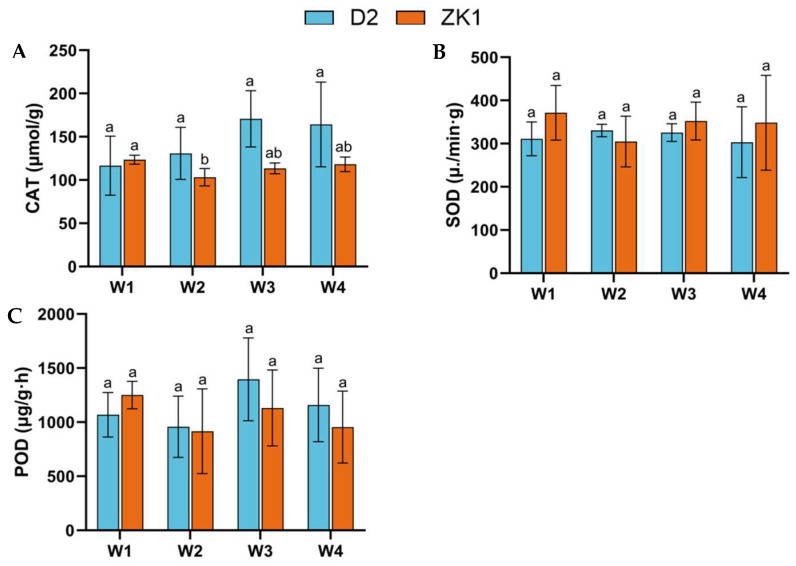
Effects of drought stress on the activities of antioxidant enzymes in quinoa varieties with different drought tolerance. (**A**) CAT, (**B**) SOD, and (**C**) POD. Data are expressed as the means ± SD of three independent experiments. Different letters indicate statistically significant differences (*p* < 0.05) among treatments.

The activity of catalase (CAT) in D2 (Figure 3A) showed a trend of an initial increase, followed by a decrease with increasing stress levels, peaking at stress W3, which was an increase of 46.52% compared with W1. In contrast, CAT activity steadily decreased in ZK1. The effect of drought stress on superoxide dismutase (SOD) and peroxidase (POD) was insignificant (Figure 3B,C). In D2, SOD activity first increased and then decreased, whereas in ZK1, SOD activity decreased under W2 stress but increased under W3 and W4 stress. Compared with W1, POD activity in D2 decreased under W2, but increased under W3 and W4. Compared with W1, POD activity in ZK1 showed a decreasing trend with decreases of 26.95%, 9.55%, and 23.69% under W2, W3, and W4 stress, respectively.

### 2.4. Standardization and Basic Analysis of Metabolomic Data

**Figure 4:**
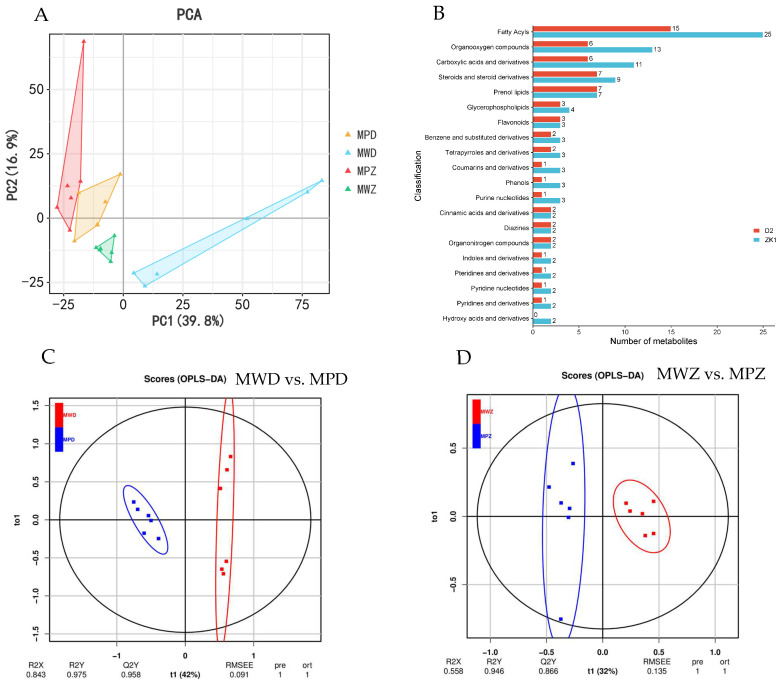
Analysis of metabolomic data under drought stress. (**A**) Principal component analysis. (**B**) Qualitative analysis. (**C**) OPLS-DA scores of MWD vs. MPD. (**D**) OPLS-DA scores of MWZ vs. MPZ.

The raw data were collected by Progenesis QI 2.0 for peak extraction and peak alignment, and then identified using the METLIN database and Biomarker Technologies Company’s library. A total of 1295 metabolites in the positive ion mode (pos) (Table S1) and 914 metabolites in the negative ion mode (neg) (Table S2) were obtained from 24 quinoa leaf samples, and the positive and negative ions were analyzed in a comprehensive manner, and partial least squares discriminant analysis (PCA-DA) was carried out on two quinoa materials, namely, D2 and ZK1, to detect the overall distribution between samples and the degree of dispersion between groups. The results of the study showed that the first principal component (PC1) and the second principal component (PC2) explained 39.8% and 16.9%, respectively, of the total variance of all the data (Figure 4A), and the results indicated that the samples were well dispersed under the two conditions, and that the data of the metabolomic analysis were reliable.

Qualitative analysis of the metabolites revealed that all metabolites were classified into 49 types. the major components were fatty acyls, organooxygen compounds, carboxylic acids and derivatives, steroids and steroid derivatives, and prenol lipids (Figure 4B).

As shown in Figure 4, significant segregation was observed in all treatments under drought stress. In the OPLS-DA plot, the cumulative values of R2Y and Q2 were 0.975 and 0.958 for D2 (Figure 4C) and 0.946 and 0.866 for ZK1 (Figure 4D), respectively, which indicated that the OPLS-DA model was not overfitted, was highly reliable and reproducible, and could be used for subsequent analysis.

### 2.5. Differential Accumulation of Metabolites in Response to Drought Stress

**Figure 5:**
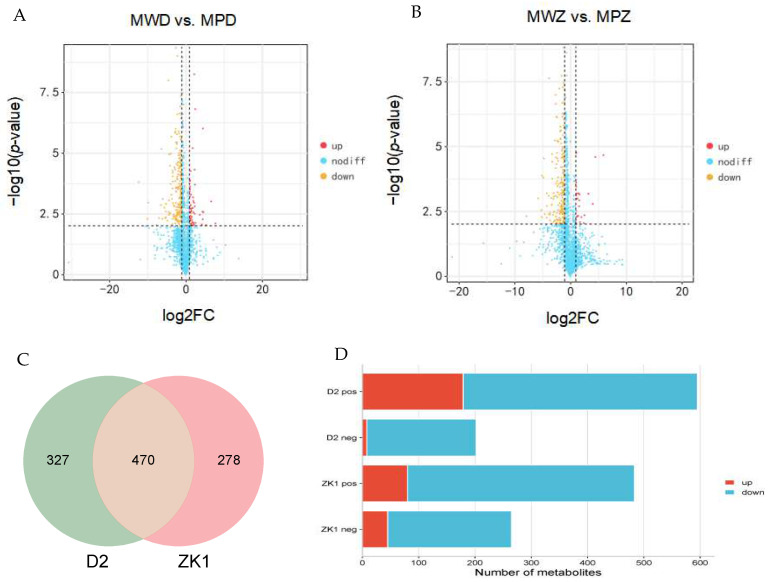
Overview of differentially accumulating metabolites. (**A**) Volcano map of the metabolites of MWD vs. MPD. (**B**) Volcano map of the metabolites of MWZ vs. MPZ. Red dots represent up-regulated metabolites, green dots represent down-regulated metabolites, and gray dots represent metabolites with no significance. (**C**) Venn diagrams of DAMs among treatments. (**D**) Up-regulation of DAMs and down-regulation of DAMs.

**Figure 6:**
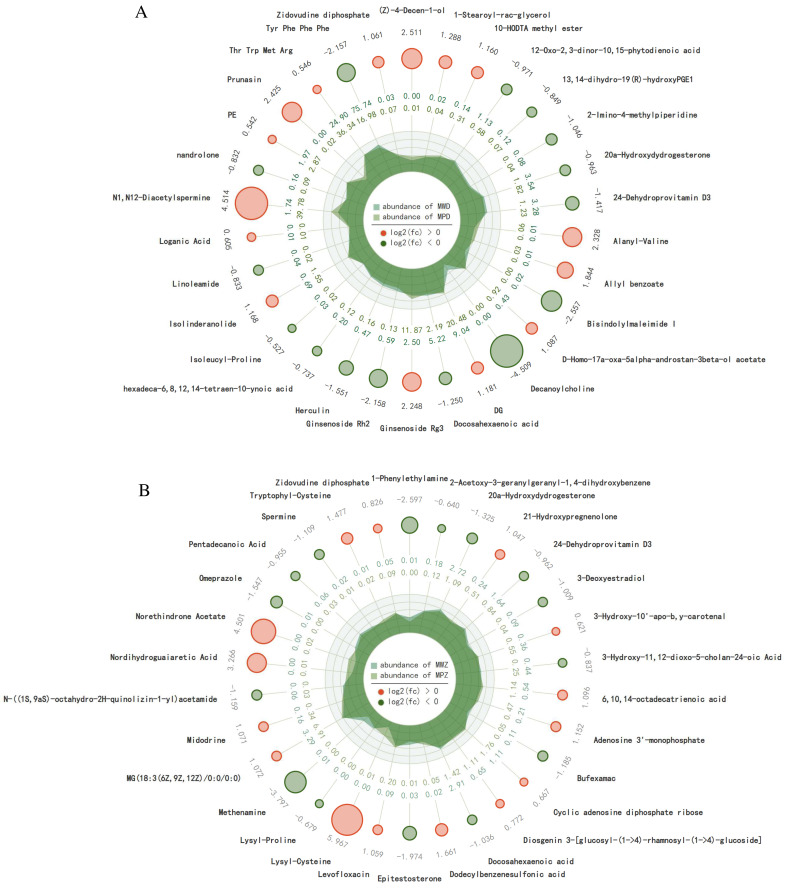
Top 15 up-regulated (red) and 15 down-regulated (green) metabolites in MPD and MPZ (**A**,**B**). The first circle of numbers indicates the log2(fc) of up-regulated accumulated metabolites and down-regulated accumulated metabolites, and the size of the circle varies with the log2(fc) value. In the second circle, the outer circle values (mean metabolite abundance of MWD × 10^3^), and the inner circle values (mean metabolite abundance of MPD × 10^3^), irregular shapes indicate the expression abundance of each group of samples.

Differentially accumulating metabolites (DAMs) were identified using the following criteria: fold change ≥ 2 and FDR < 0.01. A total of 797 DAMs (187 up-regulated and 610 down-regulated) were detected in the comparison between MWD and MPD (Figure 5A,D); the top 30 up-regulated and down-regulated DAMs are presented in Figure 6A.

A total of 748 DAMs (125 up-regulated and 623 down-regulated) were detected in the comparison between MWZ and MPZ (Figure 5B,D). The top 30 up-regulated and down-regulated DAMs are presented in Figure 6B.

Under drought stress, two experimental materials, MPD and MPZ, identified 470 metabolites (Figure 5C), with DAMs showing down-regulation > up-regulation. The number of down-regulated metabolites was ZK1 (623) > D2 (610), and the number of up-regulated metabolites was D2 (187) > ZK1 (125). The numbers of up-regulated metabolites were consistent with the strength of the tested materials’ tolerance to drought stress.

### 2.6. Annotation and Enrichment Analysis of Metabolites in the KEGG Database

**Figure 7:**
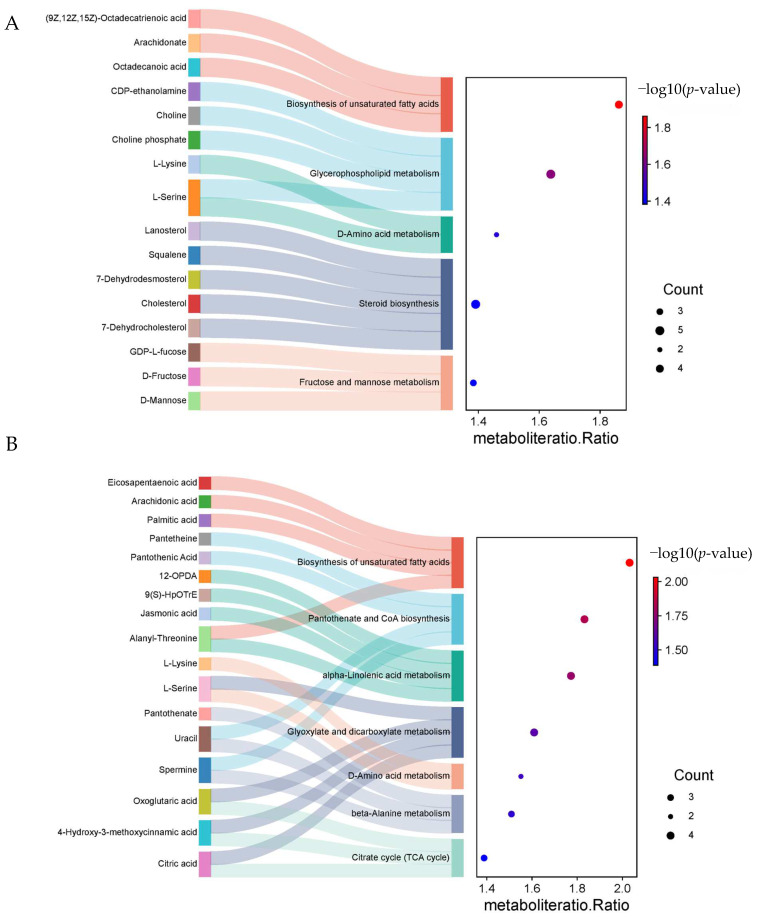
KEGG enrichment of DAMs under drought stress (**A**,**B**). The Sankey diagram illustrates the annotated metabolites within the pathways. The bubble chart displays enriched pathways, where the size of the bubbles represents the count of metabolites, and the color of the bubbles indicates the *p*-value.

When 751 DAMs from D2 were annotated with the KEGG database, mapping 58 pathways, 5 pathways were significantly enriched (*p* < 0.05) (Figure 7A). These pathways were as follows: biosynthesis of unsaturated fatty acids (ko01040), glycerophospholipid metabolism (ko00564), D-amino acid metabolism (ko00470), steroid biosynthesis (ko00100), and fructose and mannose metabolism (ko00051). The metabolites enriched in these five pathways were octadecanoic acid, arachidonate, (9Z,12Z,15Z)-octadecatrienoic acid, choline phosphate, choline, CDP-ethanolamine, L-serine, L-lysine, D-mannose, D-fructose, GDP-L-fucose, 7-dehydrocholesterol, cholesterol, 7-dehydrodesmosterol, squalene, and lanosterol, all the above metabolites were down-regulated.

When 700 DAMs from ZK1 were annotated with the KEGG database, mapping 60 pathways, 7 pathways were significantly enriched (*p* < 0.05) (Figure 7B), specifically including biosynthesis of unsaturated fatty acids (ko01040), pantothenate and CoA biosynthesis (ko00700), alpha-linolenic acid metabolism (ko00592), glyoxylate and dicarboxylate metabolism (ko00630), D-amino acid metabolism (ko00470), beta-alanine metabolism (ko00410), and citrate cycle (TCA cycle) (ko00020). A total of 17 DAMs were enriched in these 7 pathways. Oxoglutaric acid, citric acid, and 4-hydroxy-3-methoxycinnamic acid were significantly enriched in the TCA cycle pathway, all showing up-regulation.

The KEGG pathways significantly enriched in both cultivated varieties include biosynthesis of unsaturated fatty acids and D-amino acid metabolism. Specifically, D2 separately enriches the fructose and mannose metabolism pathway (ko00051), while ZK1 separately enriches the pantothenate and CoA biosynthesis pathway (ko00700) and the TCA cycle (ko00020). These pathways may play a significant role in quinoa’s response to drought stress.

## 3. Discussion

### 3.1. Phenotypes, Physiological, and Biochemical Indexes

Under drought stress, plant growth is firstly inhibited, and biomass allocation can be used as one of the criteria to measure the drought tolerance of crops [37]. Drought causes a decrease in leaf water content, water potential, osmotic potential, and chlorophyll content in maize leaves [38]. Alfalfa can maintain a high root shoot ratio under drought stress, and stress has a greater impact on root growth [39]. Quinoa has high root plasticity and leaf adaptability. In this study, drought stress significantly affected the different morphological, physiological, and biochemical characteristics of quinoa. Under mild stress (W2), the biomass of D2 was higher than the control (W1). Growth indicators showed that mild drought stress (W2) increased the growth of D2, which may be due to the harsh environment and arid climate of the Andean region, where quinoa originates and where quinoa is better adapted to survive in extreme environments.

Plants with high photosynthetic efficiency are usually more drought-tolerant [40]. Chloroplasts are the photosynthetic organelles of green plants; chlorophyll absorbs and translocates light energy and converts carbon dioxide into carbohydrates. Chloroplast content is closely linked to photosynthetic intensity and dry matter accumulation [41]. It is generally recognized that plants with a high chlorophyll content are more tolerant of drought [42]. Plants improve photosynthetic efficiency and stress tolerance by regulating chlorophyll content [43]. Drought stress causes a significant decrease in chlorophyll content in drought-sensitive wheat and a significant increase in chlorophyll content in drought-tolerant wheat [44]. The over-expression of the GKL gene in the rice variety Kitaake activates genes that increase the synthesis of chlorophyll, leading to an improvement in photosynthesis and yield [45]. The transcription factor WRYK plays a positive regulatory role in drought stress. Overexpression of MdWRYK17 in transgenic apple plants resulted in increased chlorophyll levels and enhanced drought tolerance [46], and transgenic MbWRKY2, MbWRKY3 tobacco showed enhanced CAT, APX, SOD, and POD activities, and increased chlorophyll content in response to drought stress [11,13]. The ABA signaling gene (PYL) and the chlorophyll metabolism genes (CRD1, CHLH) may be important in regulating photosynthesis and action in perilla [47]. In our study, drought stress increased total chlorophyll content, photosynthetic rate, stomatal conductance, and water use efficiency in the drought-tolerant quinoa D2, which is consistent with Liu’s [48] findings.

### 3.2. Lipid Metabolism

Lipids in plants, including lipids, membrane lipids, photosynthetic pigments, are involved in energy storage, formation of the framework of biofilm systems, signal transduction, etc., and play an important role in tolerance to biotic and abiotic stresses. According to their function, they are classified as structural and storage lipids. Palmitic, oleic, linoleic, and linolenic acids are the main components of membrane lipids, while the other lipids are mainly found in storage lipids. Under stress, fatty acid metabolism in plants undergoes complex changes [49]. Highly unsaturated fatty acids account for about 70% of all plant thylakoid membrane fatty acids. Free radicals from photosynthetic light reactions lead to the oxidation of polyunsaturated fatty acids. Unsaturated fatty acids can reduce the damage caused by drought and salt stress in plants [50]. Drought stress promotes the accumulation of fatty acid content in tomato fruits [51]. In our study, several unsaturated fatty acids were significantly increased in D2, namely (z)-6-octadecadienoic acid, (5Z, 8Z, 10e, 14z)-(12R)-12 glycolic-5,8,10,14-tetraenoic acid (12 (R)-HETE), and neuronic acid. The increased content of unsaturated fatty acids may be associated with the level of tolerance in quinoa, which may be one of the reasons for the high tolerance of quinoa.

Plants modify the stability and fluidity of cell membranes by regulating the fatty acid (FA) content and lipid composition of biological membranes, thereby resisting adverse environments. Thylakoid membranes are the site of plant photosynthesis. When plants suffer water stress, the thylakoid membranes will change the structure and material composition of the membrane according to changes in the external environment. Phospholipids consist of phosphatidylcholine (PC), phosphatidylethanolamine (PE), phosphatidylglycerol (PG), phosphatidylserine (PS), phosphatidylinositol (PI), and phosphatidic acid (PA). Choline is the major component of PC. PA is a phospholipid messenger that is important in the reorganization of the cytoskeleton and in the biosynthesis of membrane lipids [52,53]. The increase in phosphatidylethanolamine (PE) makes the cell membrane harder, and the choline in phosphatidylcholine (PC) makes the membrane more fluid. The stability of PC/PE and membrane proteins is therefore related. When the PC/PE ratio is high, it can protect the membrane from degradation [54,55]. PA and choline decreased in D2 and ZK1, while phosphatidylcholine increased in D2. D2 may promote phosphatidylcholine synthesis to maintain cell membrane integrity under drought stress. Lysophosphatidylcholine (LPC) and lysophosphatidylethanolamine (LPE) are formed from PC and PE. High lysophospholipid concentrations cause cell death. Our study found that D2 phosphatidylcholine was up-regulated, suggesting that D2 may help maintain the cell membrane structure. Monogalactose diester (MGDG) is a major component of lipids in chloroplast thylakoid membranes and is essential for maintaining the stability of membrane structure and function. MGDG plays an important role in the response to biotic and abiotic stresses and can be used as a signaling substance in response to stress. Under salt stress, increased levels of galactolipids such as MGDG may help to maintain the composition and function of photosynthetic membranes [56]. Higher ratios of PC/PE and DGDG/MGDG were seen in salt-tolerant rice than in salt-sensitive rice [57]. In this study, the content of MGDG was significantly increased in D2, while no change in the expression of this substance was found in the drought-sensitive variety ZK1. Combined with the measurement of photosynthetic indexes at the seedling stage, it was found that the photosynthetic rate, respiration rate, stomatal conductance, and WUE of drought-tolerant D2 were significantly higher than those of the drought-sensitive variety ZK1, indicating that an important regulatory mechanism of quinoa’s drought tolerance may be related to the regulatory function of the chloroplast thylakoid membranes.

### 3.3. Carbohydrate Metabolism

**Figure 8:**
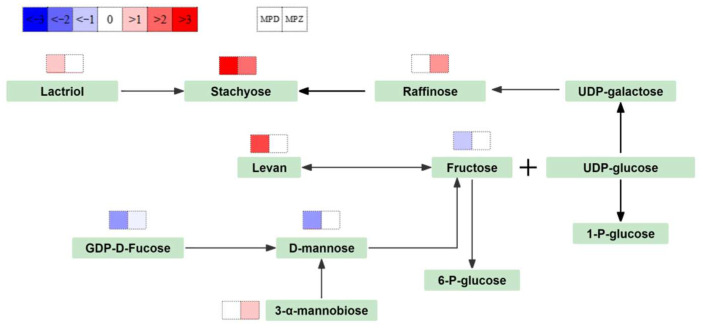
Sugar metabolism network of quinoa under drought stress. Note: Red represents metabolite up-regulation, and blue represents metabolite down-regulation.

Carbohydrate metabolism in plants plays a key role in energy storage, osmoregulation, cell recognition, and signaling molecules. When drought stress occurs, plants accumulate soluble sugars, such as sucrose, glucose, galactose, fructose, etc. Soluble sugars provide energy to plants under stress, while simultaneously enhancing their water retention capacity by maintaining cell membrane integrity and regulating osmotic pressure [58]. During drought, starch degrades into soluble sugars [59]. In our study, drought stress enhanced the starch and sucrose metabolism pathways in D2 (Figure 8), up-regulated the expression of fructooligosaccharides, N-acetyl-d-galactosamine, N-acetyl-d-galactosamine, and lactitol, and down-regulated d-fructose.

Drought enhances galactose synthesis in D2. Stachyose and raffinose are linked by sucrose and galactose. These oligosaccharides can store carbon, scavenge ROS, protect PSII, and maintain osmotic potential. Overexpression of oswrky11 in rice induced raffinose accumulation and improved drought tolerance [37]. In our study, stachyose was up-regulated in D2 and ZK1, while raffinose was up-regulated in ZK1.

Acetyl-CoA is an intermediate product of the TCA cycle, which undergoes various enzymatic reactions to produce NADPH and FAD, releasing a large amount of ATP. In our study, citric acid (1.05), ketoglutaric acid (0.62), and two types of malic acid (0.78, 0.67) were up-regulated and expressed in ZK1. Under drought stress, ZK1 may enhance the citric acid cycle to produce more energy and organic acids. During photosynthesis, the supply of NADP is crucial for the redox balance of chloroplasts, and NADP^+^ receives electrons from the photolysis of water to generate NADPH and fix CO_2_. While NADH as a precursor is catalyzed by NAD kinase to synthesize NADP^+^ [60], Shin suggested that the source of NADP^+^ is mainly from ab initio synthesis rather than the Calvin cycle [61], and NADP^+^ was found to be up-regulated in D2 (6.70) and ZK1 (3.54, 1.43), suggesting that the energy metabolism of photosynthesis plays an important role in quinoa under drought stress. It has been suggested that NAD is involved in the synthesis of ABA and proline under salt stress [62]. Whether the same mechanism exists under drought stress in quinoa needs to be further investigated.

## 4. Materials and Methods

### 4.1. Plant Materials and Growth Conditions

On the basis of the results of previous experiments, we selected the D2 (drought-tolerant) and ZK1 (drought-sensitive) varieties as experimental materials. D2 was provided by Gansu Academy of Agricultural Sciences (GAAS), and ZK1 was provided by Crop Research Institute of Chinese Academy of Agricultural Sciences (CRIAC).

Quinoa seeds were surface sterilized in a 10% sodium hypochlorite solution for 15 min and then soaked in sterile water for 6 h. The nutrient soil and perlite were mixed at a ratio of 3:1. Quinoa seeds were evenly sown in plastic flowerpots with a diameter of 10 cm and a height of 8 cm, and covered with 1 cm of nutrient soil. They were placed in a light incubator (light for 14 h; light intensity of 6000 lux; day and night temperatures of 26 °C and 20 °C, respectively; relative humidity of 60%). Each pot was watered with 200 mL twice a day. In order to ensure normal growth, Hoagland nutrient solution was watered once a week. When the seedlings grew to eight leaves, they were intercropped, and four plants were planted in each pot.

After 28 days of sowing, 12 pots of each variety were randomly divided into four groups, each group containing three pots as three replicates. The seedlings were rinsed with sterile distilled water and placed into different concentrations of PEG-6000 to simulate drought stress. The control group (W1) was treated with Hoagland nutrient solution, and the treatment group was treated with 25% (*w*/*v*), 30% (*w*/*v*), and 35% (*w*/*v*) PEG-6000 to simulate mild (W2), moderate (W3), and severe (W4) drought stress, respectively. Hoagland nutrient solution was replenished at 8 o’clock every morning to ensure that the concentration of PEG-6000 remained constant. After 10 days of stress, physiological and biochemical indexes were measured. Metabolomic analysis was performed on seedlings of the control (W1) and medium stress (W3) groups. The third leaf on the top of each seedling was fully expanded, rapidly frozen in liquid nitrogen, and stored at −80 °C. Six replicates were used for metabolomic analysis (Table 1).

### 4.2. Growth Analysis and Determination of Chlorophyll Pigments

#### 4.2.1. Determination of Seedling Growth Indicators

After 10 days of stress, 10 quinoa plants were randomly selected from each treatment, and the plant height was measured with a meter ruler. The leaf area of the top four leaves was measured with a leaf area meter (YMJ-B, TOP Holding Co., Ltd., Hangzhou, China), and the measurement data were recorded. After the measurement, we carefully took out the root of the plant, removed the root soil, and were careful not to damage the root. After cutting, we washed the aboveground and underground parts of the plant separately, dried it with filter paper, weighed the fresh weight with an electronic balance (Sartorius ME254S, Sartorius AG, Gottingen, Germany), placed it in an air blast drying oven for 30 min at 105 °C, reduced the temperature to 80 °C and dried it to constant weight, recorded the dry weight, and calculated the biomass.

#### 4.2.2. Determination of Leaf Chlorophyll Content

The total chlorophyll content of the leaves under drought stress was determined. The leaves of plants grown for 4 weeks were dried at 80 °C overnight, then 30 mg of the leaves was weighed and added to liquid nitrogen, ground, and crushed; 6 mL of 95% (*v*/*v*) ethanol was added, and the leaves were kept in the dark for 48 h. The supernatant extract was collected by centrifugation at 12,000 r/min at 4 °C for 5 min, and the absorbance of the extract at 649 nm and 665 nm was measured with a spectrophotometer (SP-752, Shanghai Spectrum Instruments Co., Shanghai, China). We calculated the chlorophyll content according to the formula:

Chl (mg/g FW) = (18.08 × OD649 + 6.63 × OD665) × 0.006/0.03

### 4.3. Oxidative Stress Indicators and Proline Content Measurement

#### 4.3.1. Determination of Leaf Photosynthetic Characteristic Indicators

After 10 days of drought stress, three quinoa plants with consistent growth were randomly selected. The net photosynthetic rate (Pn), transpiration rate (Tr), intercellular CO_2_ concentration (Ci), stomatal conductance (Gs), and water use efficiency (WUE) of the main stem and four leaves were measured during 9:30–11:00 a.m. on clear days (PP SYSTEMS CIRAS-3, Amesbury, MA, USA).

#### 4.3.2. Determination of Antioxidant Enzyme Activity

Catalase (CAT) activity was measured by the UV absorption method [63]. Superoxide dismutase (SOD) activity was measured by the nitroblue tetrazolium (NBT) method [64]. Peroxidase (POD) activity was determined by the guaiacol method [65]. Selected metabolites were further annotated with the KEGG database (http://www.kegg.jp/kegg/compound/, accessed on 21 September 2024). Annotated metabolites were then mapped to the KEGG pathway database (https://www.kegg.jp/kegg/pathway.html, accessed on 21 September 2024). 

### 4.4. Metabolomic Analysis

#### 4.4.1. Metabolite Extraction and Identification

For this procedure, 50 mg of ultra-low-temperature quinoa leaf samples was taken, then 1000 μL of an extraction solution containing an internal standard (methanol–acetonitrile–water volume ratio = 2:2:1; internal standard concentration, 2 mg/L) was added and vortexed for 30 s. Magnetic beads were added, treated with a 45 Hz grinder for 10 min, and sonicated in an ice water bath for 10 min. After standing at 20 °C for 60 min, the sample was centrifuged at 12,000 rpm for 15 min, 500 μL of supernatant was taken out in a clean centrifuge tube, and the extract was dried in a vacuum concentrator. To the dried metabolites, 160 μL of the extraction solution (acetonitrile–water volume ratio = 1:1) was added, vortexed for 30 s, sonicated in ice water bath for 10 min, and centrifuged at 12,000 rpm for 15 min at 4 °C. Next, 120 μL of the supernatant was taken off into a 2 mL injection bottle, 10 μL of each sample was taken, and these were mixed into QC samples for machine detection.

The data acquisition instrument system included ultra performance liquid chromatography (Waters UPLC Acquity I-class plus) and high-resolution mass spectrometry (Waters UPLC Xevo G2-xs QTof), and the selected chromatographic column (Acquity UPLC HSS T3 1.8 μm 2.1 × 100 mm, Waters Corporation, Milford, MA, USA).

#### 4.4.2. LC-MS Data Analysis

The raw data collected with masslynx v4.2 were processed by Progenesis Qi 2.0 for peak extraction, peak alignment, and other data processing operations. The identification was carried out based on the online metlin database (https://metlin.scripps.edu/, accessed on 21 September 2024) of Genesis Qi software and the self-built database of BmK. At the same time, the theoretical fragments were identified, and the mass number deviation was within 100 ppm.

SIMCA V18.0 was used to perform unsupervised principal component analysis (PCA) and orthogonal projections to later structures-discriminant analysis (OPLS-DA) analysis of normalized data. For biological replicates, the differential metabolites were screened by combining the difference multiple, the *p*-value of the t-test, and the VIP value of the OPLS-DA model. The screening criteria were FC > 1, *p*-value < 0.05, and VIP > 1.

### 4.5. Statistical Analysis

Physiological data were organized using Excel. One-way ANOVA was performed and plotted using GraphPad Prism 10.3.1 software. Significant differences were detected using Duncan’s test (*p* < 0.05). Metabolomic KEGG data enrichment analysis was performed using metaboanalyst 6.0.

## 5. Conclusions

In this study, the effects of drought stress on quinoa seedlings were investigated at phenotypic, physiological, and metabolomic levels using quinoa with different drought tolerance. The results showed that drought stress increased the biomass of drought-tolerant quinoa and improved the chlorophyll content, photosynthetic rate, and water use efficiency. Metabolomics results showed that the unsaturated fatty acid pathway and glycerophospholipid pathway were significantly enriched, and the differential metabolites 12(R)-HETE, phosphatidylcholine, MGDG, and stachyose were up-regulated under drought stress. In conclusion, quinoa responds to drought stress by accumulating chlorophyll and sugar, activating unsaturated fatty acid metabolism, and protecting the photosynthetic system.

## Figures and Tables

**Table 1:** Composition of treatments at the quinoa seeding stage.

Plant Material	Treatment	LC-MS Samples
D2	Water	MWD
	Drought	MPD
ZK1	Water	MWZ
	Drought	MPZ

## Data Availability

The raw data supporting the conclusions of this article will be made available by the authors, without undue reservation.

## Supplementary Materials

The following supporting information can be downloaded at https://www.mdpi.com/article/10.3390/ijms26062599/s1.
